# Editorial: Cell Signaling Mediating Critical Radiation Responses

**DOI:** 10.3389/fonc.2021.695355

**Published:** 2021-05-10

**Authors:** Carsten Herskind, Mary Helen Barcellos-Hoff

**Affiliations:** ^1^ Department of Radiation Oncology, Universitätsmedizin Mannheim, Medical Faculty Mannheim, Heidelberg University, Mannheim, Germany; ^2^ Department of Radiation Oncology, University of California, San Francisco, San Francisco, CA, United States

**Keywords:** radiotherapy, radiation response, cell signaling, microenvironment, DNA repair, hypoxia, immunotherapy, fibrosis

Radiotherapy is an important loco-regional component of modern multimodal cancer therapy. Classically, radiobiology’s 5 Rs: repair, repopulation, redistribution, reoxygenation, and radiosensitivity, as described by Steele et al. (1) have informed the use of radiotherapy. The past several decades have filled in molecular mechanism by which the radiation response of cells and tissues affect tumor control, expanding our knowledge of survival pathways beyond repair of radiation damage to DNA (2). In many cases, these pathways receive cues from extracellular signaling molecules *via* membrane-bound receptors (3). This emphasizes the importance of both intra- and extracellular signaling, and the interaction between different cell types in radiation-induced cell death as well as normal-tissue reaction after radiotherapy (4–6). The articles in the present Research Topic discuss recent progress and present original research on the contribution of cell signaling to radiation responses and radiation effects on cell signaling.

Residual double-strand breaks and complex DNA lesions are considered lethal and characteristic for ionizing radiation although typically some 98% are repaired by non-homologous end joining (NHEJ; all cell-cycle phases) or homologous recombination (HR, which is restricted to late S- and G2 phase). While the yield of single-strand breaks and base damage is much higher, these lesions contribute little to cell killing under normal circumstances as they are repaired with very high efficiency by the base excision repair (BER) system, although some single strand breaks in S-phase may be converted to double-strand break at replication forks. If HR is compromised (e.g. by *BRCA1* mutation), or overloaded, an error-prone backup mechanism, alternative end joining (alt-EJ) will repair the lesions. Liu et al. reviews the significant regulation of repair pathway choices by an extracellular cytokine, transforming growth factor (TGFβ), whose activity is a prominent signal in the irradiated tumor. TGFβ promotes HR and NHEJ but suppresses alt-EJ (7), and thus blocking TGFβ signaling increases radiosensitivity. An intracellular route to shifting repair competency is reviewed by Starcher et al. who describe the rationale for using quinone substrates to induce futile redox cycles leading to oxidative damage, BER hyperactivation, and depletion of NAD+ and ATP, which synergizes with ionizing radiation to radiosensitize human cancer cells at moderate doses. The study by Shukla et al. explores the synergy of combining the quinone derivative, β-lapachone with inhibition of a mitochondrial enzyme involved in folate metabolism.

Tumor hypoxia is well-recognize factor associated with resistance to radiotherapy and other therapies. The review by Sørensen and Horsman focuses on the hypoxia-inducible factor (HIF) regulatory pathway, stress responses, and inflammatory pathways, and discusses the clinical applications of vascular modifiers and biomarkers. Extracellular signaling *via* transmembrane receptors can influence repair and survival pathways, and inhibition of EGFR is established in the clinic in combination with radiation therapy of head and neck, non-small cell lung and other cancers (8). Many cytokines, hormones and some growth factors activate the transcription factor STAT3 which is also involved in radioresistance as discussed by Wang et al. The study by Zhang et al. shows that a so far poorly characterized protein, FAM53A, influences vital cell functions related to the MAP kinase pathway in a p53-dependent manner and may be a candidate for targeted therapy in combination with DNA-damaging agents.

Radiotherapy is the canonical example of a DNA damage agent whose benefit is ascribed to cell kill in the irradiated field yet more than 15 years ago Formenti and Demaria showed that radiation could elicit systemic response in combinations with immunotherapy and formalized the concept by describing radiation as an ‘*in situ* vaccination’ that can synergize with immunotherapy (9–11). Even though so-called ‘abscopal’ response, i.e., outside the irradiated field, are rare in the clinic, the idea of radiotherapy creating an ‘in situ’ vaccine has engendered significant interest given the remarkable successes of immune checkpoint blockade and other immunotherapy in many cancer patients. The study by Chen et al. on experimental hepatocarcinoma irradiated with different fractionation schemes found anti-tumor effects upon rechallenge with a reduction in immunosuppressing cells. Irradiation of the hepatocarcinoma inoculated on hind limbs triggered the antitumor immunity capable of suppressing the growth of subcutaneous tumors, accompanied with reduced myeloid derived suppressor cells in both blood and tumors. Radiotherapy can play a part in enhancing the response to immunotherapy by releasing damage-associated molecular pattern (DAMP) molecules that activate pattern recognition receptors (PRR, e.g. toll-like receptors, TLR), thereby acting as an immunological adjuvant (11). Domankevich et al. show that a PRR agonist synergizes with radiation from α-emitters in the treatment of experimental tumors.

In addition, complex signaling effects to and from the immune system beyond activation of immune cells are also beginning to be recognized. The study by Xia et al. describes how a small-molecule inhibitor of PD-L1 can radiosensitize glioblastoma cells by downregulating MiR-33a causing upregulation of PTEN, an endogenous suppressor of the AKT survival pathway. The clinical study by Minnaar et al. on mild hyperthermia in addition to chemoradiotherapy in cervical cancer patients found a marked increase in complete metabolic resolution in lymph nodes outside the irradiated field at six months.

The microenvironment of tumors and the surrounding stroma are key factors for both tumor control and normal tissue toxicities. Farias et al. review the evidence for the ability of mesenchymal stem cells and signaling *via* exosomes to mediate bystander (i.e., near-neighbor) and abscopal effects of radiotherapy. The study by Meyer et al. describes how host CD39 (part of the purinergic signaling pathway that responds to purine derivatives such as ATP and adenosine) has suppressive effects on the growth and radiosensitivity of Lewis lung tumors, which was abrogated in CD39 knockout mice and was associated with effects on the endothelial compartment. At the same time, CD39 deficiency enhanced radiation-induced lung fibrosis and osteopontin expression. Subcutaneous fibrosis after breast radiotherapy affects patients’ quality of life and is an endpoint for risk prediction and mechanisms of radiation-induced fibrosis (12). Liu et al. review phosphatidic acid-mediated signaling from diacylglycerol kinase alpha and identify roles in T-cell activation, exosome production, and cell-cycle regulation as potential targets for interference. Finally, Herskind et al. analyzed genes expression profiles and genetic pathways associated with radiation-induced cell-cycle arrest and differentiation of fibroblasts *in vitro*. Notably, upregulated inflammatory pathways provide a potential link between fibrotic reaction and the immune system.

## Outlook

Therapeutic resistance is often intrinsic to the cancer cell, but signals generated between cells in the tumor microenvironment may ultimately determine response or resistance to radiotherapy, underscoring a gap that hampers cancer treatment optimization. Together, the papers in this Research Topic emphasize the importance of physiological context and intercellular communication within tumors and tissue as well as systemic signaling. Several provide a strong rationale for manipulating these signals that could enable optimization of radiotherapy.

## Author Contributions

All authors contributed to the article and approved the submitted version.

## Conflict of Interest

The authors declare that the research was conducted in the absence of any commercial or financial relationships that could be construed as a potential conflict of interest.
